# Supplementary material for: Rural children remain more at risk of acute malnutrition following exit from community based management of acute malnutrition program in South Gondar Zone, Amhara Region, Ethiopia: a comparative cross-sectional study
Source: PeerJ. 2020 Feb 7;8:e8419. doi: 10.7717/peerj.8419 (PMC7008819; doi:10.7717/peerj.8419)
Supplement: Supplemental Information 2 [file peerj-08-8419-s002.pdf]

Table: Factors associated with acute malnutrition of children age 6-59 mothers following discharge as recovered in South Gondar Zone, Amhara region, Ethiopia, 2017/18

Variables (recovered)	Acute malnutrition			95% CI		95%CI
	Yes	No	P-Value	COR	P-Value	AOR
**Place of district**						
Ebnat	125 (51.4)	176 (37.9)	<0.0001	4.26 (2.31-7.85)	0.001	3.31 (1.66-6.61)
Tach Gayint	104 (42.8)	204 (44.0)	<0.0001	3.06 (1.66-5.65)	0.012	2.38 (1.21-4.69)
Lay Gayint	14 (5.8)	84 (18.1)		1		1
**Religion**						
Orthodox	221(90.9)	436 94.0()		1		1
Muslim	22 (9.1)	28 (6.0)	0.139	1.55 (0.87-2.77)	0.618	1.21(0.57-2.55)
**HH on Food Aid**						
No	167 (68.7)	351 (75.6)		1		1
Yes	76 (31.3)	113 (24.4)	0.049	1.41(1.00-1.99)	0.713	1.08 (0.71-1.65)
**Currently on Family Planning**						
No	66 (27.2)	77 (16.6)	0.001	1.87 (1.29-2..72)	0.274	1.28 (0.82-1.99)
Yes	177 (72.8)	387 (83.4)		1		1
**Got an education on child feeding**						
No/don't remember	60 (25.1)	80 (17.2)	0.013	1.61 (1.10-2.36)	0.702	1.10(0.68-1.76)
Yes	182 (74.9)	384 (82.8)		1		1
**Attend ANC**						
**No**	56 (23.0)	87 (18.8)	0.178	1.30(0.89-1.90	0.986	1.01 (0.59-1.71)
**Yes**	187 (77.0)	377 (81.2)		1		1
**Sex of child**						
Male	133 (54.7)	228 (49.1)	0.158	1.25 (0.92-1.71)	0.041	1.43(1.01-2.01)
Female	110 (45.3)	236 (50.9)		1		1
**Prelactal feeding**						
No	215 (88.5)	445 (95.9)		1		1

Yes	28 (11.5)	19 (4.1)	<0.0001	3.05 (1.67-5.59)	0.008	2.56 (1.28-5.12)
**Prepare food separately from the family**						
No	124 (51.0	152 (32.8)	<0.0001	2.14 (1.56-2.94)	0.857	0.96 (0.64-1.45)
Yes	119 (49.0)	312 (67.2)		1		1
**Respondent education**						
Non formal	213 (87.7)	367 (79.1)	0.005	1.88(1.21-2.92)	0.531	1.19 (0.69-2.05)
Formal	30 (12.3)	97 (20.9)		1		1
**Respondent Occupation**						
Farming	185 (76.1)	388 (83.6)		1		1
Other than farming	58 (23.9)	76 (16.4)	0.016	1.60 (1.09-2.35)	0.307	1.26 (0.81-2.00)
**HH family size**						
>=5	148 (60.9)	252 (54.3)	0.09	1.31 (0.96-1.80)	0.587	1.13 (0.73-1.73)
<5	95 (39.1)	212 (45.7)		1		1
**Vitamin A supplement past 6 months**						
No/don't remember	91 (37.4)	80 (17.2)	<0.0001	2.87 (2.02-4.10)	0.000	2.13(1.42-3.19)
Yes	152 (62.6)	384 (82.8)		1		1
**Vaccinated for measles**						
No/don't remember	14 (5.8)	15 (3.2)	0.112	1.83 (0.87-3.86)	0.120	1.91 (0.85-4.30)
Yes	229 (94.2)	449 (96.8)		1		1
**Deworming given past 6 months**						
No/don't remember	200 (82.3)	357 (76.9)	0.098	1.39 (0.94-2.07)	0.854	1.05 (0.64-1.74)
Yes	43 (17.7)	107 (23.1)		1		1
**Duration after recovery in a month**						
<=3	74 (30.5)	160 (34.5)	0.034	0.62 (0.39-0.97)	0.554	0.84(0.48-1.49)
4-6	115 (47.3)	232 (50.0)	0.052	0.66 (0.44-1.00)	0.498	0.84(0.51-1.39)
7-9	54 (22.2)	72 (15.5)		1		1
**HH food secure**						
No	80 (32.9)	84 (18.1)	0.000	2.22 (1.55-3.17)	0.815	1.06(0.66-1.70)
Yes	163 (67.1)	380 (81.9)		1		1
**Decision maker on seek care to sick child**						

Together (Husband and wife)	158 (65.0)	358 (22.8)		1		1
Either(Husband or Wife)	85 (35.0)	106 (77.2)	0.013	1.61 (1.10-2.35)	0.506	1.15 (0.76-1.75)
**Dry waste disposal**						
Open field	147 (60.5)	213 (45.9)	0.000	1.80 (1.32-2.47)	0.058	1.48 (0.99-2.21)
Put into pin/burn	96 (39.4)	251 (54.1)		1		1
**Child feces disposal**						
Unsafe	99 (40.7)	220 (47.4)		1		1
Safe	144 (59.3)	244 (52.6)	0.091	1.31 (0.96-1.80)	0.003	1.73 (1.21-2.48)
**History of cough (URTI) past 2 weeks**						
No	229 (94.2)	448 (96.6)				1
Yes	14 (5.8)	16 (3.4)	0.152	1.71 (0.82-3.57	0.314	1.58 (0.65-3.83)
**Time to water source (round trip)**						
<30 minutes	88 (36.2)	192 (41.4)		1		
30 minutes or longer	155 (63.8)	272 (58.6)	0.183	1.24 (0.90-1.71)	0.320	1.20 (0.83-1.72)
**Additional food during pregnancy/ lactation**						
No	124 (51.0)	151(32.5)	<0.0001	2.16 (1.57-2.97)	0.013	1.58 (1.10-2.28)
Yes	119 (49.0)	313(67.5)		1		1
**Colostrum feeding**						
No	82 (33.7)	109 (23.5)	0.004	1.66 (1.18-2.34)	0.046	1.51 (1.01-2.26)
Yes	161 (66.3)	355 (76.5)		1		1
**BF initiation within an hr. after birth**						
No	99 (40.7)	161(34.7)	0.114	1.29 (0.94-1.78)	0.973	1.01 (0.63-1.61)
Yes	144 (59.3)	303 (65.3)		1		1
**Age of HH head in years**						
<=36	111 (45.7)	266 (57.3)		1		1
>36	132 (54.3)	198 (42.7)	0.003	1.60 (1.17-2.18)	0.866	1.04 (0.63-1.73)
**Age of respondent in years**						
<=30	97 (39.9)	245(52.8)		1		1
>30	146 (60.1)	219 (47.2)	0.001	1.68 (1.23-2.31)	0.007	1.63 (1.15-2.31)

**Birth order**						
1-2	87 (35.8)	208 (44.8)		1		1
3+	156 (64.2)	256 (55.2)	0.021	1.44(1.06-2.01)	0.663	1.19 (0.55-2.57)
**Treatment stay in week (contentious)**			0.001	0.85 ()0.78-0.94)	0.062	0.91 (0.82-1.01)
**Wealth index**						
Lowest	37 (15.4)	68 (14.8)	0.876	0.96 (0.54-1.69)	0.110	0.58 (0.29-1.13)
Second	50 (20.8)	70 (15.2)	0.412	1.26 (0.73-2.16)	0.635	0.86 (0.46-1.61)
Middle	67 (27.9)	183 (39.8)	0.078	0.64 (0.39-1.05)	0.074	0.60 (0.34-1.05)
Fourth	49 (20.4)	74 (16.1)	0.584	1.16 (0.68-2.00)	0.153	0.64 (0.35-1.18)
Highest	37 (15.4)	65 (14.1)		1		1

Table: Factors associated with acute malnutrition of children age 6-59 mothers never treated for acute malnutrition in South Gondar Zone, Amhara region, Ethiopia, 2017/18

Variables (comparative)	Acute malnutrition			95% CI		95%CI
	Yes	No	P-Value	COR	P-Value	AOR
**Place of district**						
Ebnat	80 (42.3)	262 (50.6)	0.089	1.69 (0.92-3.09)	0.130	1.65 (0.86-3.14)
Tach Gayint	94 (49.7)	173 (33.4)	0.000	3.01 (1.64-5.50)	0.007	2.49 (1.29-4.81)
Lay Gayint	15 (7.9)	83 (16.0)		1		1
**Religion**						
Orthodox	164 (86.8)	490 (94.6)		1		1
Muslim	25 (13.2)	28 (5.4)	<0.0001	2.67 (1.51-4.71)	0.068	1.79 (0.96-3.36)
**HH on Food Aid**						
No	120 (65.3)	370 (71.4)		1		1
Yes	69 (36.5)	148 (28.6)	0.043	1.44 (1.01-2.04)	0.439	0.84 (0.54-1.31)
**Sex of child**						
Male	113 (59.8)	270 (52.1)	0.071	1.37 (0.97-1.92)	0.037	1.47 (1.02-2.11)
Female	76 (40.2)	248 (47.9)		1		1
**Prepare food to children separately from family diet**						
No	92 (48.7)	224 (43.2)	0.199	1.25 (0.89-1.74)	0.788	1.05 (0.72-1.55)
Yes	97 (51.3)	294 (56.8)				
**Respondent education**						
Non formal	159 (84.1)	397 (76.6)	0.033	1.62 (1.04-2.51)	0.484	1.20 (0.72-1.99)
Formal	30 (15.9)	121 (23.4)		1		1
**Vit A supplement past 6 months**						
No/don't remember	88(46.6)	181 (34.9)	0.005	1.62 (1.16-2.28)	0.523	1.14 (0.76-1.71)
Yes	101 (53.4)	337 (65.1)		1		1
**Number of under-five children**						
2+	34 (18.0)	119(23.0)	0.155	1.36 (0.89-2.08)	0.351	1.24 (0.79-1.96)
1	155 (82.0)	399 (77.0)		1		1

**HH food secure**						
No	68 (36.0)	110 (21.2)	0.000	2.08 (1.45-3.00)	0.024	1.59 (1.06-2.38)
Yes	121 (64.0)	408 (78.8)		1		1
**Good hand washing practice**						
No	100 (52.9)	219 (42.3)	0.012	1.53 (1.10-2.14)	0.017	1.55 (1.08-2.21)
Yes	89 (47.1)	299 (57.7)		1		1
**Colostrum given**						
No/don't remember	74 (39.6)	129 (25.0)	0.000	1.97 (1.38-2.81)	0.010	1.66 (1.23-2.45)
Yes	113 (60.4)	388 (75.0)		1		1
**Got an education on child feeding**						
No/don't remember	54 (28.6)	123 (23.7)	0.190	1.29 (0.88-1.87)	0.115	1.40 (0.92-2.13)
Yes	135 (71.4)	395 (76.3)		1		1
**Birth interval**						
<24	31 (16.4)	51 (9.8)	0.017	1.80(1.11-2.91)	0.012	1.92 (1.15-3.19)
>=24	158 (83.6)	467 (90.2)		1		1
**Time to water source (round trip)**						
<30 minutes	75 (39.7)	234 45.2()		1		
30 minutes or longer	114 (60.3)	284 (54.8)	0.193	1.25 (0.89-1.76)	0.407	1.17 (0.81-1.71)
**Home to HP by foot walk in minute**						
<=30	83 (43.9)	300 (57.9)		1		1
>30	106 (56.1)	218 (42.1)	0.001	1.76 (1.26-2.46)	0.006	1.66 (1.16-2.37)
**Decision maker on expenditure**						
Either husband or wife	48 (25.4)	193 (37.3)	0.003	1.74 (1.20-2.53)	0.183	1.31(0.88-1.96)
Both husband and wife	141 (74.6)	325 (62.7)		1		1
